# Targeting POLRMT by a first-in-class inhibitor IMT1 inhibits osteosarcoma cell growth in vitro and in vivo

**DOI:** 10.1038/s41419-024-06444-9

**Published:** 2024-01-16

**Authors:** Yang Kong, Xiangrong Li, Huanle Zhang, Bin Fu, Hua-Ye Jiang, Hui-Lin Yang, Jin Dai

**Affiliations:** 1 Department of Orthopedics, the First Affiliated Hospital of Soochow University, Suzhou, China; 2 Department of Orthopedics, The First People’s Hospital of ChuZhou, ChuZhou, China; 3 Department of Pharmacy, Kongjiang Hospital of Yangpu District, Shanghai, China; 4 Department of Radiotherapy, Suzhou Ninth People’s Hospital, Suzhou, China; 5 Orthopedic Institute, Medical College, Soochow University, Suzhou, China; 6 Department of Orthopedics, Suzhou Wujiang District Children’s Hospital, Suzhou, China

**Keywords:** Bone cancer, Targeted therapies

## Abstract

Osteosarcoma (OS) is a highly aggressive form of bone cancer that predominantly affects adolescents and young adults. In this study, we have undertaken an investigation into the potential anti-OS cell activity of IMT1 (inhibitor of mitochondrial transcription 1), a first-in-class inhibitor of RNA polymerase mitochondrial (POLRMT). IMT1 exhibited a profound inhibitory effect on cell survival, proliferation, cell cycle progression, and migration in primary and immortalized OS cells. Furthermore, this POLRMT inhibitor elicited apoptosis in the OS cells, without, however, inducing cytotoxicity in human osteoblasts or osteoblastic cells. IMT1 disrupted mitochondrial functions in OS cells, resulting in mitochondrial depolarization, oxidative injury, lipid peroxidation, and ATP reduction in OS cells. Silencing POLRMT using targeted shRNA closely mimicked the actions of IMT1 and exerted potent anti-OS cell activity. Importantly, IMT1’s effectiveness was diminished in POLRMT-silenced OS cells. Subsequent investigations revealed that IMT1 suppressed the activation of the Akt-mammalian target of rapamycin (mTOR) cascade in OS cells. IMT1 treatment or POLRMT silencing in primary OS cells led to a significant reduction in Akt1-S6K-S6 phosphorylation. Conversely, it was enhanced upon POLRMT overexpression. The restoration of Akt-mTOR activation through the introduction of a constitutively active S473D mutant Akt1 (caAkt1) mitigated IMT1-induced cytotoxicity in OS cells. In vivo, oral administration of IMT1 robustly curtailed the growth of OS xenografts in nude mice. Furthermore, IMT1 suppressed POLRMT activity, impaired mitochondrial function, repressed Akt-mTOR activation, and induced apoptosis within xenograft tissues. Collectively, these findings underscore the potent growth-inhibitory effects attributed to IMT1 via targeted POLRMT inhibition. The utilization of this POLRMT inhibitor carries substantial therapeutic promise in the context of OS treatment.

## Introduction

Osteosarcoma (OS) is an aggressive form of bone cancer that primarily affects adolescents and young adults [1–3]. OS accounts for approximately 2–3% of all childhood cancers and the incidence is estimated to be 4–5 cases per million individuals in the United States [1–3]. The current standard treatment for OS typically involves a combination of surgery, chemotherapy and in some cases, radiation therapy [4–6]. Overall, the 5-year survival rate for localized OS is approximately 60–70%, but it drops significantly in patients with advanced/metastatic OS [1–3]. OS is characterized by various genetic alterations and molecular abnormalities. Molecularly targeted therapy is a promising avenue for OS by targeting the specific signalings vital for OS progression [7–10]. Researchers have identified several key genetic mutations and signaling pathways involved in the development and progression of OS. Some targeted therapies are being investigated, including receptor tyrosine kinase (RTK) inhibitors (i.e. sorafenib and pazopanib), mTOR inhibitors (i.e. everolimus) and insulin-like growth factor 1 receptor (IGF-1R) inhibitors (i.e. ganitumab and cixutumumab) [7–10].

The proper functioning of mitochondria and the integrity of mitochondrial DNA (mtDNA) are crucial for maintaining OS development and progression by regulating energy metabolism, reactive oxygen species (ROS) production, genomic instability and others [11–16]. A significant number of mitochondria-related genes are dysregulated in OS [11–16]. For example, the mitochondrial protein ADCK1 (AarF domain-containing kinase 1) is upregulated in OS [11]. This upregulation was found to play a crucial role in sustaining mitochondrial hyperfunction and facilitating the growth of OS cells, both in vitro and in vivo [11]. In a separate investigation, the silencing of N-myc downstream-regulated gene 1 (NDRG1) was shown to impair mitochondrial functions, induce differentiation of cancer stem cells, and mitigate the progression of OS [17].

POLRMT, or RNA polymerase mitochondrial, is a key enzyme for the transcription of mtDNA in eukaryotic cells [18–21]. Located within the mitochondria, POLRMT plays a pivotal role in the synthesis of essential mitochondrial RNA molecules, which are subsequently utilized in the production of key respiratory chain components [18–21]. These components are integral to the mitochondria’s energy-producing functions, making POLRMT indispensable for cellular energy metabolism [18–21]. Dysregulation or malfunction of POLRMT has been implicated in various mitochondrial disorders and diseases (including cancer [15, 22–24]). Zhou et al. have shown that mitochondrial expression of POLRMT is increased in human non-small cell lung cancer (NSCLC) tissues and cells, important for cancer cell growth [22]. POLRMT shRNA or knockout (through CRSIPR/Cas9 method) blocked mtDNA transcription and inhibited NSCLC cell growth [22]. Significantly, Han and colleagues revealed the overexpression of POLRMT in OS, leading to an enhancement of mitochondrial functions and the facilitation of OS cell growth [15]. Remarkably, the genetic silencing of POLRMT, accomplished through the utilization of shRNA or CRISPR/Cas9 methodologies, exerted potent inhibitory effect on the growth of OS cells [15].

Bonekamp and colleagues have recently pioneered the development of IMT1 (inhibitor of mitochondrial transcription 1) as an innovative, first-in-class inhibitor of POLRMT, demonstrating specificity and noncompetitive inhibition properties in their studies [23, 25]. IMT1 induces conformational alteration of POLRMT and effectively disrupts mitochondrial transcription, oxidative phosphorylation (OXPHOS) and mitochondrial biogenesis [23, 26]. In this study, we systematically assessed the prospective anti-OS efficacy of IMT1 and conducted an in-depth exploration of the plausible underlying mechanisms.

## Materials and methods

### Reagents, chemicals, and antibodies

Polybrene, cell culture medium, the anti-oxidant n-acetylcysteine (NAC), ATP, serum, cell counting kit-8 (CCK-8), puromycin, caspase inhibitors, and various other reagents were generously supplied by Sigma-Aldrich (St. Louis, MO). Antibodies and mRNA primers were obtained from Dr. Shi at Soochow University [15]. All fluorescent dyes were procured from Thermo-Fisher Invitrogen (Shanghai, China). IMT1 was sourced from Dr. Li [26].

### Cells

MG63 immortalized cells, along with the primary human OS cells (pOS-1, pOS-2, pOS-3, originating from primary written-informed consent patients), and primary human osteoblasts were generously contributed by Dr. Cao [27–29]. The hFOB1.19 human osteoblastic cells were sourced from Dr. Liang and cultivated following established procedures [30, 31]. To validate the phenotypes, a comprehensive monitoring regimen was established, including bi-monthly assessments for STR profiling, determination of population doubling time, and scrutiny of cell morphology. Ethical approval for the utilization of primary human cells was granted by the Ethics Committee of Soochow University, in accordance with the principles outlined in the Declaration of Helsinki.

### Cell Counting Kit-8 (CCK-8)

Cells were seeded into 96-well plates at a density of 3.5 × 10^3^ cells in 200 μL of medium per well. The cells were then incubated in a 5% CO_2_ atmosphere at 37 °C. Subsequently, 20 μL of CCK-8 solution was added to each well and further incubated for 2 h. The absorbance of the CCK-8 solution at 550 nm was quantified using a microplate reader.

### Cellar fluorescence staining experiments

Cells were placed in 24-well plates at a concentration of 3–5 × 10^4^ cells in 500 μL of medium per well and incubated for the specified duration. After incubation, the cells were fixed with 4% paraformaldehyde, followed by a PBS wash and subsequent incubation with 0.3% Triton at room temperature. Fluorescence dyes were then added to the cells, which were subsequently washed and examined under a Leica microscope. The fluorescence intensity was quantified using an ImageJ software (NIH).

### Transwell assays

For cell migration assays, Transwell chambers (Corning, NY) were employed. Briefly, the chambers were gently placed into a 24-well plate, and 600 µL of cell culture medium containing 10% serum (FBS) was added to the lower chamber. Cells were resuspended in cell culture medium without FBS, and 200 µL of this suspension (1.5 × 10^4^ cells per chamber) was added to the upper surface. After 16 h of incubation, cells on the upper surface were removed using cotton swabs, while those on the lower surface were fixed, and stained. Images were captured under a microscope.

### GSH/GSSG ratio

The quantification of the ratio between reduced glutathione (GSH) and oxidized glutathione (GSSG), serving as a reflection of the cellular redox state, was conducted using a commercially available kit (Sigma). GSH was subjected to derivatization, leading to the formation of 5,5’-dithiobis (2-nitrobenzoic acid), generating a yellow-hued compound that was subsequently assessed through spectrophotometric analysis at 450 nm. Each treatment involved the testing of twenty μL of cellular/tissue lysates.

### Assessment of mitochondrial complex I activity and ATP levels

The enzymatic activity of complex I was determined via a commercially available kit (Sigma), employing spectrophotometric techniques to monitor the conversion of NADH to NAD^+^ catalyzed by complex I. The corresponding reduction in absorbance at 400 nm was documented as a direct indicator of complex I activity. Cellular and tissue ATP levels were measured utilizing a commercial colorimetric kit (Sigma) in accordance with the provided protocols. Each treatment involved the testing of twenty μL of cellular or tissue lysates.

### Akt1 mutation

Lentiviral particles carrying the constitutively active S473D mutant Akt1 (caAkt1), generously provided by Dr. Chen [32], were introduced into cultured OS cells. The establishment of stable cells expressing caAkt1 was achieved through selective pressure using puromycin-based selection methods.

### POLRMT shRNA

Lentivirus containing human POLRMT shRNA, obtained from Dr. Shi [22] at Soochow University (Suzhou, China), was employed for gene silencing. Primary human OS cells in polybrene-supplemented complete medium were exposed to the virus at a multiplicity of infection (MOI) of 12 for a duration of 48 h. Subsequently, cells were returned to complete medium containing puromycin for the selection of stable cells, a process that continued for five passages. Control cells were transduced with lentivirus containing scramble control non-sense shRNA (“shC”). The expression of POLRMT at both the mRNA and protein levels was consistently confirmed in the established cells.

### POLRMT overexpression

Cells were subjected to infection with lentivirus carrying the POLRMT-overexpressing construct, which was provided by Dr. Zhou [22]. This construct incorporated a puromycin selection gene but did not include a GFP-Tag. Subsequently, puromycin-containing medium was introduced and maintained for an additional five passages, resulting in the establishment of stable cells. Control cells were stably transduced with an empty lentiviral vector (“Vec”).

### Other assays

The comprehensive procedures for additional assays, such as the colony formation, Caspase-3 and Caspase-9 activity assessments, apoptosis evaluations utilizing nuclear TUNEL staining, single strand DNA (ssDNA), Western blot analysis, quantitative real-time polymerase chain reaction (qRT-PCR), and cell cycle progression assessment via PI-FACS, have been thoroughly delineated in previously published studies [32–35]. Figure S1 listed all uncropped blotting images.

### Xenograft studies

Xenograft experiments were carried out employing athymic nude mice aged 4–5 weeks, with an even distribution of both male and female subjects, and a weight range of 17.7–18.1 grams. These mice were procured from the Shanghai Laboratory Animal Center (Shanghai, China). Six million pOS-1 primary cells per mouse suspended in 150 µL of Matrigel basic medium (no serum) were subcutaneously (*s.c*.) injected into these mice, resulting in the establishment of xenograft tumors with volumes approaching 100 mm³ within a span of four weeks. Following tumor establishment, the mice were segregated into two distinct groups: One group receiving oral administration of IMT1 (50 mg/kg body weight), while the other group received a vehicle control. The body weights of the mice and the volumes of the tumors [calculated using the formula V = π/6 × (larger diameter) × (smaller diameter)²] were recorded. All animal-related procedures were conducted in compliance with the ethical guidelines and regulations, with approval obtained from the Institutional Animal Care and Use Committee (IACUC) and Ethics Committee of Soochow University.

### Tissue lipid peroxidation assay

For quantification of lipid peroxidation, we employed the thiobarbituric acid reactive substances (TBAR) assay to determine the concentration of malondialdehyde (MDA). MDA reacts with thiobarbituric acid (TBA), leading to the formation of a distinct pink-colored complex. Specifically, tissue lysates (40 μg per treatment) were mixed with a previously described reaction buffer [36, 37], and the resulting mixture was subjected to boiling at 100 °C for a duration of 30 min. Subsequently, centrifugation was carried out at 3000 rpm for 12 min, and the absorbance of the resulting pink-colored solution was measured at 545 nm.

### Tissue fluorescence assay

Paraffin-embedded xenograft slides were baked, dewaxed, and hydrated. To mitigate non-specific binding, goat serum was utilized for blocking the tissue slices for a duration of 20 min at 37 °C. The tissue slices were then subjected to incubation with the specified TUNEL and DAPI fluorescence dyes, followed by a thorough wash, and subsequent examination utilizing a confocal microscope (ZEISS).

### Statistical analysis

Throughout all in vitro experiments, assessments regarding group allocations were conducted in a blinded manner. The in vitro experiments were subjected to replication on five separate biological repeats. Data exhibiting a normal distribution were expressed in terms of the mean ± standard deviation (SD). Statistical analysis was carried out employing SPSS version 22.0 (SPSS Co., Chicago, IL). In the case of comparisons involving two specific groups, the unpaired Student’s *t*-test was employed. For comparisons encompassing more than two groups, the one-way ANOVA with the Scheffe’ and Tukey Test was applied. Statistical significance was attributed to *P*-values < 0.05.

## Results

### IMT1 suppresses survival, proliferation, cell cycle progression, and motility of OS cells

**Fig. 1:**
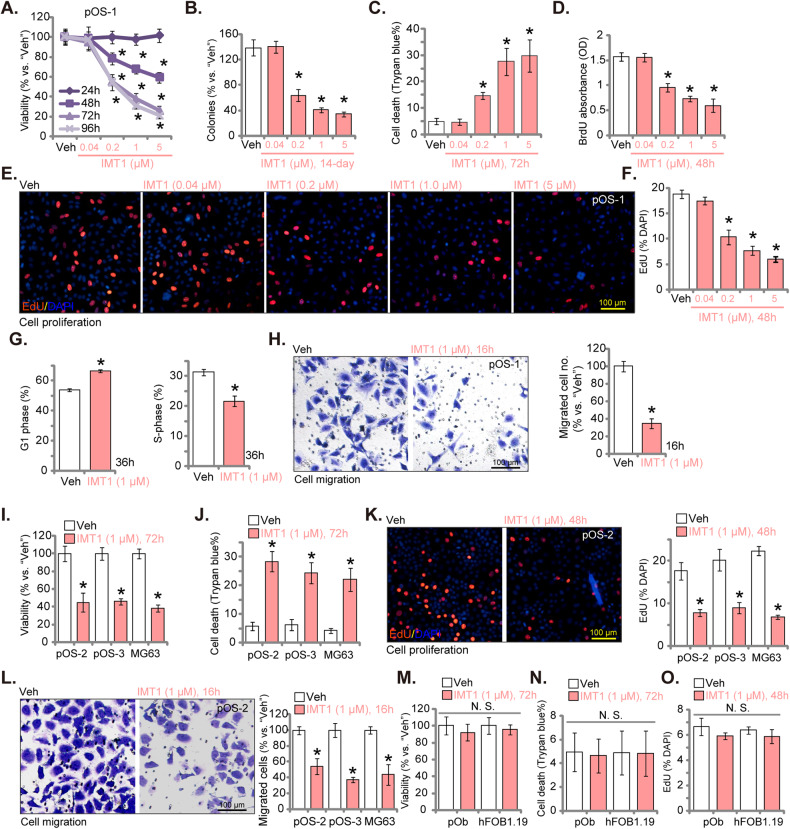
IMT1 suppresses survival, proliferation, cell cycle progression, and motility of OS cells. The primary human OS cells, pOS-1, were treated with IMT1 (at designated concentrations) for indicated time periods, cell viability (CCK-8 assay, **A**), colony formation (**B**), cell death (Trypan blue staining assay, **C**), cell proliferation (BrdU/EdU assays, **D**–**F**), cell cycle progression (PI-FACS) (**G**) and in vitro migration (“Transwell” assay, **H**) were measured via the corresponding methods, with results quantified. Other primary OS cells (pOS-2 and pOS-3) (**I**-**L**), the immortalized MG63 cells (**I**–**L**), primary human osteoblasts (“pOb”) (**M**–**O**) or hFOB1.19 osteoblastic cells (**M**–**O**) were treated with IMT1 (1 μM) for indicated time periods, cell viability (CCK-8 assays, **I** and **M**), death (Trypan blue staining assays, **J** and **N**), proliferation (EdU assays, **K** and **O**), and in vitro migration (“Transwell” assay, **L**) were measured via the corresponding methods, with results quantified. “Veh” designates the vehicle control group. *P < 0.05 indicates a significant difference compared to the “Veh” treatment. “N. S.” represents *P* > 0.05, indicating no statistical difference. The data is presented as mean ± SD. The experiments presented in this figure were replicated five times (*n* = 5, with five biological replicates), yielding consistent and similar results. The scale bar is set at 100 μm.

The primary human OS cells, pOS-1 [29], were treated with different concentrations of IMT1 (0.04/0.2/1/5 μM) and were further cultivated for 24-96 h. Cell viability was thereafter measured by CCK-8 assay. IMT1 dose-dependently suppressed viability of pOS-1 cells (Fig. 1A). At 0.2-5 μM, the POLRMT inhibitor significantly decreased CCK-8 viability of pOS-1 cells (Fig. 1A). Moreover IMT1 decreased pOS-1 cell viability in time-dependently and it took at least 48 h for the POLRMT inhibitor (0.2-5 μM) to exert a significant effect (Fig. 1A). Following treatment of IMT1 (0.2-5 μM), the number of pOS-1 cell colonies was robustly decreased (Fig. 1B). The POLRMT inhibitor induced significant death of pOS-1 cells and it dose-dependently increased the number of cells with positive Trypan blue staining (Fig. 1C). IMT1 also efficiently inhibited pOS-1 cell proliferation. Bromodeoxyuridine (BrdU) absorbance (Fig. 1D) and EdU-positive nuclei ratio (Fig. 1E, F) were both significantly decreased after IMT1 (0.2–5 μM) treatment. It should be pointed out that the low dose of IMT1, 0.04 μM, did not significantly alter cell viability (Fig. 1A), colony formation (Fig. 1B), cell death (Fig. 1C) and proliferation (Fig. 1D–F). Titration experiments revealed that a concentration of 1 μM of IMT1 exhibited potent anti-OS cell activity, as illustrated in Fig. 1A–F. Given its proximity to the half-maximal inhibitory concentration (IC-50), this concentration was chosen for subsequent experiments.

Further, IMT1 (1 μM) disrupted cell cycle progression and induced G1-S arrest in pOS-1 cells (Fig. 1G). After treatment with the POLRMT inhibitor, the proportion of cells in G1 phase increased significantly but the proportion of cells in S phase decreased (Fig. 1G). Our investigation also emphasized the impact of IMT1 on the motility of pOS-1 cells. As demonstrated, treatment with IMT1 (1 μM) largely inhibited pOS-1 cell in vitro migration (Fig. 1H), assessed using “Transwell” assays. In the migration experiments, cells were exposed to IMT1 for a duration of 16 h (Fig. 1H), which did not have any adverse effects on cell viability or induce cell death (Fig. 1A, C).

Subsequently, we investigated the potential impact of IMT1 on other OS cells, including primary human OS cells derived from two additional patients (pOS-2 and pOS-3) as well as immortalized MG63 cells. Treatment with 1 μM of IMT1 prominently suppressed cell viability and led to a reduction in CCK-8 optical density (OD) in both primary and immortalized OS cells (Fig. 1I). Moreover, IMT1 treatment induced significant cell death, as evidenced by an increased Trypan blue staining intensity in OS cells (Fig. 1J). Furthermore, the proliferation rate, determined by the ratio of EdU-positive nuclei, was also hindered by the POLRMT inhibitor (Fig. 1K). The “Transwell” assays provided additional support for the inhibitory effects of IMT1 on the in vitro migration of both primary and immortalized OS cells (Fig. 1L).

The potential activity of IMT1 on non-cancerous cells, including hFOB1.19 osteoblastic cells and primary human osteoblasts (“pOb”, provided by Dr. Cao [27]), was studied. Unlike in OS cells, treatment with IMT1 (1 μM) failed to decrease cell viability (CCK-8 OD, Fig. 1M), induce cell death (Fig. 1N) and inhibit cell proliferation (EdU incorporation, Fig. 1O) in hFOB1.19 cells and primary osteoblasts. The above results confirm the specific effect of the POLRMT inhibitor in OS cells.

### IMT1 induces apoptosis activation in OS cells

**Fig. 2:**
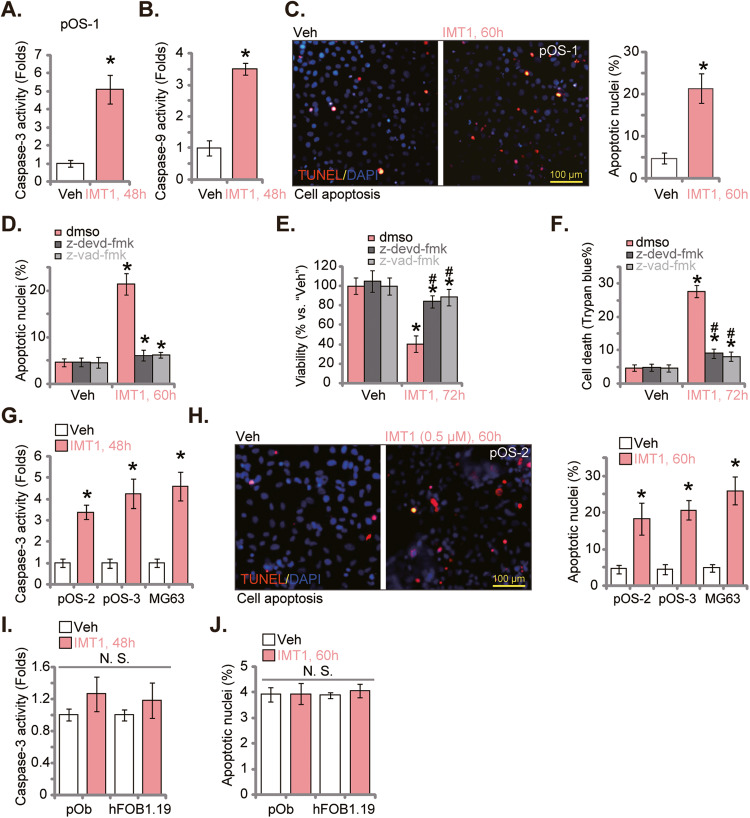
IMT1 induces apoptosis activation in OS cells. The primary human OS cells, pOS-1, were treated with IMT1 (1 μM) for indicated time periods, the Caspase-3 activity (**A**) and the Caspase-9 activity (**B**) were tested; Cell apoptosis was examined via nuclear TUNEL staining (**C**). pOS-1 cells were pretreated for 1 h with the Caspase-3 specific inhibitor z-devd-fmk (50 μM), and the pan caspase inhibitor z-vad-fmk (50 μM) or the 0.2% DMSO control (“dmso”), followed by IMT1 (1 μM) treatment for indicated time periods, cell apoptosis, viability and death were tested by nuclear TUNEL staining (**D**), CCK-8 (**E**) and Trypan blue staining (**F**) assays, respectively. Other primary human OS cells (pOS-2 and pOS-3) (**G**, **H**), the immortalized MG63 cells (**G**, **H**), primary human osteoblasts (“pOb”) (**I**, **J**) or hFOB1.19 osteoblastic cells (**I**, **J**) were treated with IMT1 (1 μM) for indicated time periods, the Caspase-3 activity was measured (**G**, **I**). Cell apoptosis was examined via nuclear TUNEL staining (**H**, **J**) assays. “Veh” designates the vehicle control group. *P < 0.05 indicates a significant difference compared to the “Veh” treatment. ^#^ *P* < 0.05 versus “dmso” group (**E**, **F**). “N. S.” represents *P* > 0.05, indicating no statistical difference. The data is presented as mean ± SD. The experiments presented in this figure were replicated five times (*n* = 5, with five biological replicates), yielding consistent and similar results. The scale bar is set at 100 μm.

The potential effect of IMT1 on apoptosis in OS cells was studied next. In pOS-1 primary cells, treatment with the POLRMT inhibitor (1 μM) robustly enhanced the Caspase-3 activity (Fig. 2A) and the Caspase-9 activity (Fig. 2B). Significant pOS-1 cell apoptosis was detected after IMT1 stimulation. The TUNEL-positively stained nuclei ratio (Fig. 2C) was substantially increased following IMT1 treatment. Two caspase inhibitors, including the Caspase-3 specific inhibitor z-devd-fmk and the pan Caspase inhibitor z-vad-fmk, almost blocked IMT1 (1 μM)-induced apoptosis in pOS-1 cells (Fig. 2D). The two inhibitors also largely ameliorated IMT1 (1 μM)-induced pOS-1 cell viability reduction (Fig. 2E) and cell death (Fig. 2F). Therefore, apoptosis induction should be a key mechanism of IMT1-induced cytotoxicity of OS cells.

In other primary OS cells (pOS-2 and pOS-3) and immortalized MG63 cells, treatment with IMT1 (1 μM) significantly increased the Caspase-3 activity (Fig. 2G). In these OS cells, significant apoptosis was observed after IMT1 treatment, evidenced by increased TUNEL-positively stained nuclei (Fig. 2H). Contrarily, in hFOB1.19 osteoblastic cells and primary human osteoblasts (“pOb”), same IMT1 (1 μM) treatment failed to increase the Caspase-3 activity (Fig. 2I) and TUNEL-positively stained nuclei (Fig. 2J). These experimental results further confirm the specific activity of IMT1 in OS cells.

### IMT1 disrupts mitochondrial functions in OS cells

**Fig. 3:**
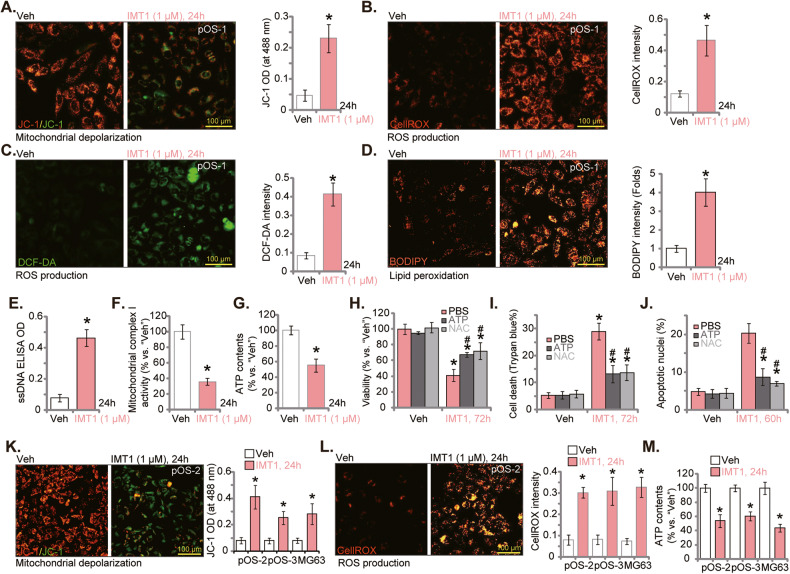
IMT1 disrupts mitochondrial functions in OS cells. The primary OS cells, pOS-1, were treated with IMT1 (1 μM) for 24 h, mitochondrial depolarization was analyzed by JC-1 staining (**A**); ROS production was tested via CellROX and DCF-DA dye assays (**B**, **C**), with lipid peroxidation examined via BODIPY staining (**D**); The ssDNA contents were measured via ELISA assay (**E**); The mitochondrial complex I activity (**F**) and ATP contents (**G**) were also measured. pOS-1 cells were pretreated for 1 h with NAC (500 μM), ATP (1 mM) or PBS, followed by IMT1 (1 μM) treatment for indicated time periods, cell viability, death and apoptosis were tested by CCK-8 (**H**), Trypan blue staining (**I**) and nuclear TUNEL staining (**J**), assays, respectively. Other primary OS cells (pOS-2 and pOS-3) or the immortalized MG63 cells were treated with IMT1 (1 μM) for 24 h, mitochondrial depolarization (JC-1 dye assay, **K**), ROS production (CellROX dye assay, **L**) and ATP contents (**M**) were tested similarly. “Veh” designates the vehicle control group. *P < 0.05 indicates a significant difference compared to the “Veh” treatment. ^#^ *P* < 0.05 versus “PBS” group (**H**–**J**). The data is presented as mean ± SD. The experiments presented in this figure were replicated five times (*n* = 5, with five biological replicates), yielding consistent and similar results. The scale bar is set at 100 μm.

The potential effect of IMT1 on the mitochondrial functions was studied. As shown, in pOS-1 primary cells, IMT1 (1 μM) stimulation for 24 h caused depolarization of mitochondria, which was evidenced by the conversion of JC-1 red fluorescence aggregates to green fluorescence monomers [38, 39] (Fig. 3A). Significant ROS production and oxidative injury were detected in IMT1 (1 μM)-treated pOS-1 cells. The CellROX red fluorescence intensity (Fig. 3B) and the DCF-DA (2’,7’-dichlorofluorescein diacetate) green fluorescence intensity (Fig. 3C) were both significantly increased. Moreover, IMT1 induced lipid peroxidation in pOS-1 primary cells and the BODIPY (difluoroborondipyrromethene) fluorescence intensity was increased (Fig. 3D). Oxidative injury shall induce DNA damage and the single strand DNA (ssDNA) contents were indeed increased (Fig. 3E). Importantly, the mitochondrial complex I activity was inhibited (Fig. 3F) and the ATP contents were decreased (Fig. 3G) in pOS-1 cells after IMT1 (1 μM) treatment.

There above results supported that IMT1 disrupted mitochondrial functions in OS cells. Importantly, the antioxidant NAC and exogenously added ATP mitigated IMT1-induced cytotoxicity. Viability reduction (Fig. 3H), cell death (Fig. 3I) and apoptosis (Fig. 3J) by IMT1 were alleviated after NAC or ATP pre-treatment. IMT1 disrupted mitochondrial functions in other OS cells. In pOS-2/pOS-3 primary cells and MG63 cells, IMT1 (1 μM) induced mitochondrial depolarization (Fig. 3K), ROS production (Fig. 3L) and ATP depletion (Fig. 3M).

### IMT1 is ineffective in POLRMT-silenced OS cells

**Fig. 4:**
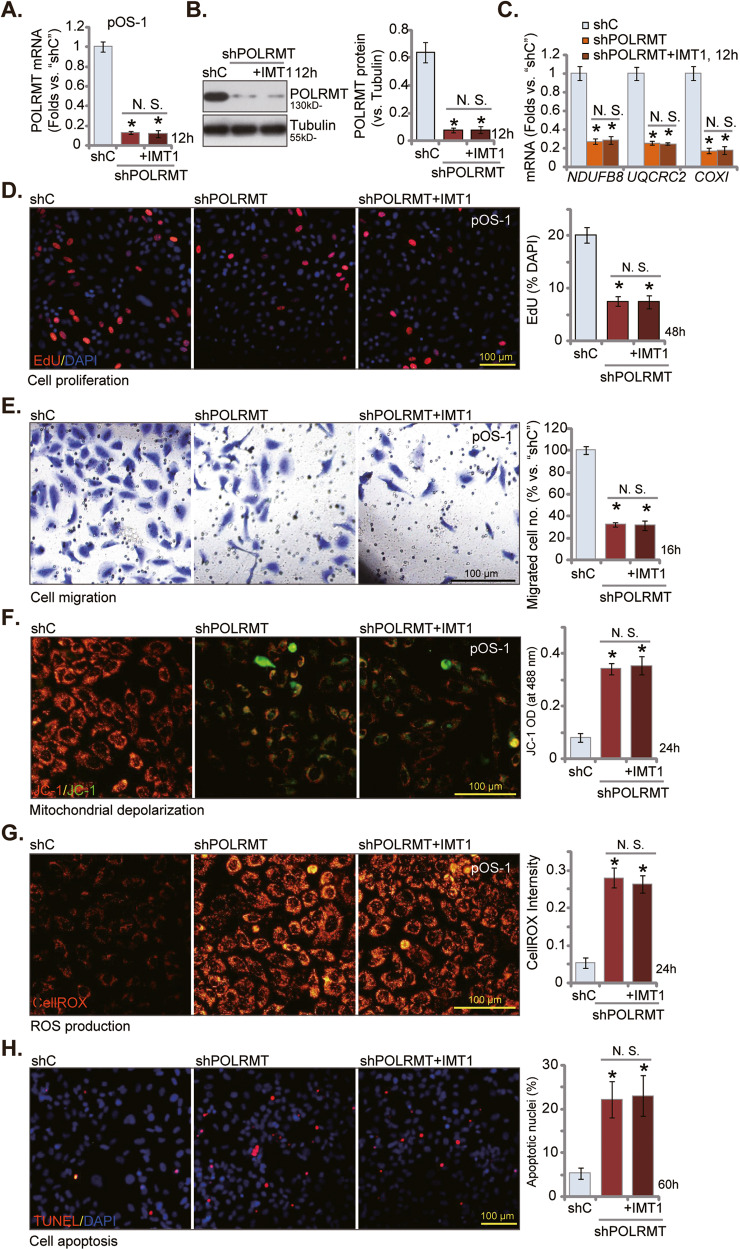
IMT1 is ineffective in POLRMT-silenced OS cells. The stable primary pOS-1 cells with the lentiviral POLRMT shRNA (“shPOLRMT”) were treated with or without IMT1 (1 μM) for indicated time periods, control cells with the scramble control shRNA (“shC”) were left untreated. Expression of listed mRNAs and proteins was shown (**A**–**C**); Cell proliferation (EdU dye assay **D**) and in vitro migration (“Transwell” assay, **E**) were measured via the corresponding methods, with results quantified; Mitochondrial depolarization was analyzed by JC-1 staining (**F**); ROS production was tested via CellROX dye assay (**G**), with cell apoptosis examined by TUNEL-nuclei staining assay (**H**). *P < 0.05 indicates a significant difference compared to the “shC” treatment. “N. S.” represents *P* > 0.05, indicating no statistical difference. The data is presented as mean ± SD. The experiments presented in this figure were replicated five times (*n* = 5, with five biological replicates), yielding consistent and similar results. The scale bar is set at 100 μm.

In the event that the anti-OS cellular response elicited by IMT-1 were attributed to the inhibition of POLRMT, its efficacy would be anticipated to be nullified in cells with depleted POLRMT. To verify this hypothesis, shRNA strategy was employed to silence POLRMT. Specifically, the lentiviral particles containing POLRMT shRNA sequence (“shPOLRMT”) were added to pOS-1 cells, and stable cells established after puromycin-based selection. Expression of *POLRMT* mRNA (Fig. 4A) and protein (Fig. 4B) was indeed substantially decreased in shPOLRMT pOS-1 cells. POLRMT-dependent mitochondrial transcripts, including *NDUFB8*, *UQCRC2,* and *COXI* [15, 22, 23, 26, 40], were also downregulated in POLRMT-silenced pOS-1 cells (Fig. 4C). Importantly, adding IMT1 failed to alter expression of POLRMT and POLRMT-dependent genes in pOS-1 cells (Fig. 4A–C).

Mimicking IMT1-induced actions, POLRMT silencing inhibited proliferation (EdU incorporation, Fig. 4D) and migration (Fig. 4E) of pOS-1 cells. POLRMT shRNA impaired mitochondrial functions and induced mitochondrial depolarization, evidenced by JC-1 green monomers accumulation (Fig. 4F). Oxidative stress was also observed in POLRMT-silenced pOS-1 cells, which was supported by CellROX fluorescence intensity increase (Fig. 4G). Apoptosis was induced in shPOLRMT-expressing pOS-1 cells, with TUNEL-positive nuclei ratio significantly increased (Fig. 4H). Importantly, adding IMT1 failed to further inhibit proliferation (EdU incorporation, Fig. 4D) and migration (Fig. 4E) of shPOLRMT pOS-1 cells. Neither did it induce more mitochondrial dysfunction (Fig. 4F, G) and apoptosis (Fig. 4H). Therefore, IMT1 was ineffective in POLRMT-silenced OS cells.

### IMT1 inhibits Akt-mTOR cascade activation in OS cells

**Fig. 5:**
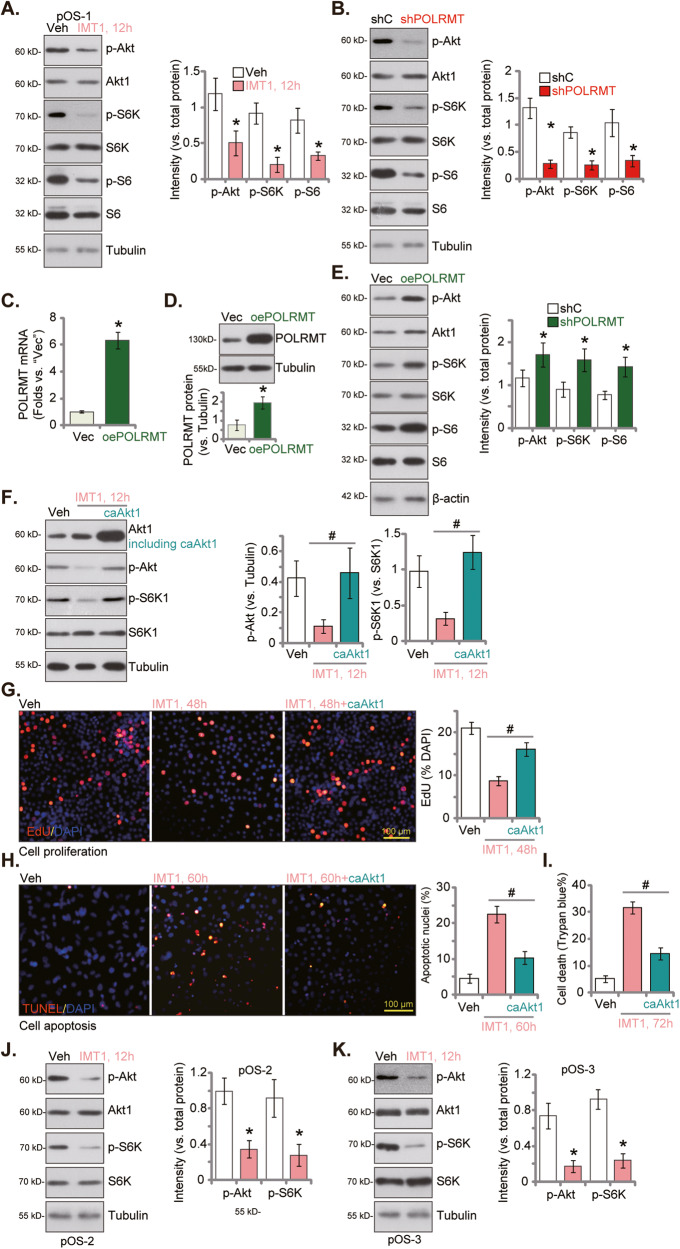
IMT1 inhibits Akt-mTOR cascade activation in OS cells. The primary OS cells, pOS-1, were treated with IMT1 (1 μM) for 12 h, listed proteins in total cell lysates were tested (**A**); The stable primary pOS-1 cells with the lentiviral POLRMT shRNA (“shPOLRMT”) or the scramble control shRNA (“shC”) were cultivated for 12 h, listed proteins in total cell lysates were tested (**B**); The stable pOS-1 cells with the lentiviral POLRMT-expressing construct (“oePOLRMT”) or empty vector (“Vec”) were cultivated for 12 h, *POLRMT* mRNA (**C**) and listed proteins in total cell lysates (**D**, **E**) were tested; The stable pOS-1 cells with constitutively active S473D mutant Akt1 (caAkt1) were treated with IMT1 (1 μM) for designated hours, and control cells were treated with vehicle control (“Veh”), listed proteins in total cell lysates were tested (**F**); Cell proliferation, apoptosis and death were examined by EdU-nuclei staining (**G**), TUNEL-nuclei staining (**H**) and Trypan blue staining (**I**) assays, respectively. The primary pOS-2 cells (**J**) or pOS-3 cells (**K**) were treated with IMT1 (1 μM) for 12 h, listed proteins in total cell lysates were tested. “Veh” stands for vehicle control. *P < 0.05 indicates a significant difference compared to “Veh”/“shC”/“Vec” (**A**–**E**, **J** and **K**). ^#^ ***P*** < 0.05 indicates a significant difference (**G**–**I**). The data is presented as mean ± SD. The experiments presented in this figure were replicated five times (*n* = 5, with five biological replicates), yielding consistent and similar results. The scale bar is set at 100 μm.

Akt-mTOR is key signaling pathway that plays a significant role in the development and progression of OS [10, 41–44]. Mitochondrial hyperfunction can foster Akt-mTOR activation in cancer cells [45–47]. We next tested whether IMT1 altered Akt-mTOR cascade in OS cells. In pOS-1 primary cancer cells, treatment with IMT1 (1 μM) potently inhibited phosphorylation of Akt (Ser-473), ribosomal protein S6 kinase 1 (S6K, Thr-389) and S6 (Ser-235/236) (Fig. 5A), indicating Akt-mTOR inactivation. Total Akt1, S6K, and S6 expression was not significantly changed (Fig. 5A). In addition, shRNA-induced silencing of POLRMT (“shPOLRMT”, see Fig. 4) also inhibited Akt-mTOR cascade activation and decreased Akt-S6K-S6 phosphorylation in pOS-1 cells (Fig. 4B). Expression of Akt1, S6K and S6 was again intact in shPOLRMT cells (Fig. 5B). Contrarily, we show that ectopic overexpression of POLMRT increased Akt-mTOR activation in OS cells. pOS-1 cells was transduced with a lentiviral POLRMT-expressing construct and stable cells (“oePOLRMT”) was established after puromycin-initiated selection. The mRNA (Fig. 5C) and protein (Fig. 5D) expression of POLMRT was substantially increased in oePOLRMT pOS-1 cells. Consequently, Akt-S6K-S6 phosphorylation was augmented (Fig. 5E), where total Akt1, S6K and S6 were unchanged (Fig. 5E).

To explore the role Akt-mTOR inactivation in IMT1-induced anti-OS cell activity, a lentivirus-packed constitutively active S473D mutant Akt1 (caAkt1) was stably transduced to pOS-1 cells (Fig. 5F), and it restored Akt-S6K phosphorylation in IMT1-treated cells (Fig. 5F). Functional, IMT1-induced proliferation inhibition (EdU-nuclei reduction, Fig. 5G), cell apoptosis (TUNEL-nuclei increasing, Fig. 5H) and death (Trypan blue assays, Fig. 5I) were dramatically alleviated by caAkt1. In other primary OS cells, pOS-2 and pOS-3, treatment with IMT1 (1 μM) for 12 h similarly inhibited Akt-S6K phosphorylation, without affecting total Akt1-S6K expression (Fig. 5J. K). These results indicated that Akt-mTOR inactivation is important for IMT1-induced cytotoxicity in OS cells.

### IMT1 administration inhibits OS xenograft growth in nude mice

**Fig. 6:**
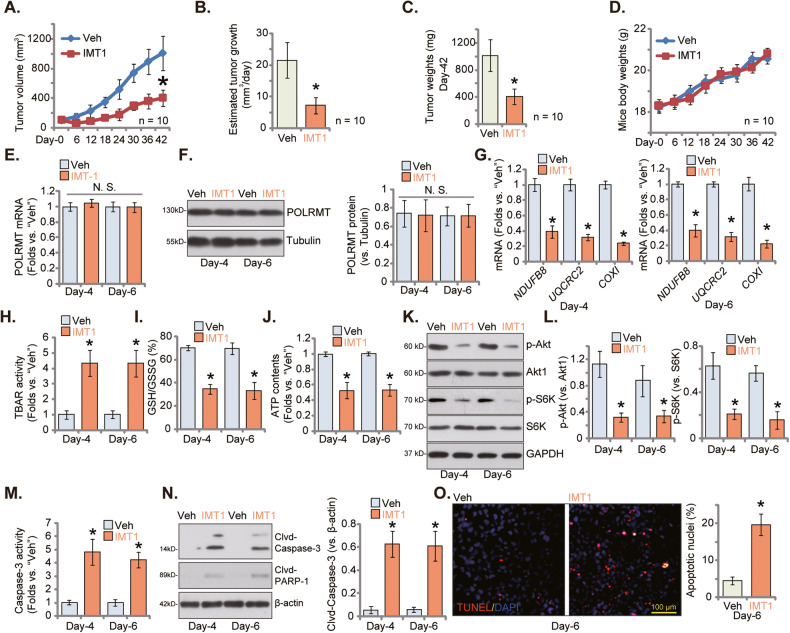
IMT1 administration inhibits OS xenograft growth in nude mice. The nude mice harboring pOS-1 xenografts were subjected to oral administration of IMT1 at a dose of 50 mg/kg body weight, with treatments administered every 48 h (for four founds), while the control group received vehicle (“Veh”). At six-day intervals, the volumes of pOS-1 xenograft tumors (**A**) and the body weights of the animals (**D**) were recorded. To assess the rate of tumor growth, the estimated daily increase in tumor volume in mm^3^ per day was computed (**B**). On “Day-42,” all mice were humanely euthanized by decapitation and the xenograft tumors were meticulously isolated and weighed (**C**). On Days 4 and 6, precisely 12 h following the administration of either IMT1 or the vehicle, one pOS-1 xenograft from each group was isolated, and the expression levels of the specified genes and proteins in the pOS-1 xenograft tissues were analyzed (**E**–**G**, **K**, **L** and **N**). In addition, measurements were taken to determine TBAR intensity (**H**), the GSH/GSSG ratio (**I**), ATP levels (**J**) and Caspase-3 activity (**M**). Alternatively, pOS-1 xenograft slices underwent immunofluorescence staining of TUNEL-positive nuclei (**O**). The data is presented as mean ± SD. In **A**–**D**, ten mice were in each group (*n* = 10). For **E**–**O**, five random tissue pieces in each xenograft were tested (*n* = 5). *P < 0.05 indicates a significant difference compared to vs. “Veh” group. “N. S.” represents *P* > 0.05, indicating no statistical difference. The scale bar is set at 100 μm.

Finally, we conducted the in vivo testing to evaluate the potential anti-OS cell activity of IMT1. pOS-1 cells were subcutaneously (*s.c*.) injected into the flanks of nude mice, resulting in the formation of pOS-1 xenografts after a four-week incubation period (labeled as “Day-0”). Subsequently, the xenograft-bearing mice were randomly divided into two groups: one receiving IMT1 treatment and the other serving as a control group receiving the vehicle control (“Veh”). IMT1 was administered at a dose of 50 mg/kg body weight, administered every 48 h for a total of four rounds (on Day-0, Day-2, Day-4, and Day-6) [26]. The tumor growth curve data presented in Fig. 6A clearly illustrates the potent inhibitory effect of IMT1 on the growth of pOS-1 xenografts in nude mice. The volumes of pOS-1 xenografts in the IMT1 treatment group were significantly lower compared to those in the vehicle control group, as depicted in Fig. 6A. To further assess the impact, we employed a previously established formula [27, 29] to calculate the estimated daily tumor growth rate in mm^3^ per day. The results, as shown in Fig. 6B, confirm that IMT1 administration effectively suppressed pOS-1 xenograft growth and significantly reduced daily tumor growth. At Day-42, all pOS-1 xenografts from both groups were meticulously isolated and individually weighed. Notably, the pOS-1 xenografts in the IMT1-treated group exhibited significantly lower weight compared to those in the vehicle group (Fig. 6C). Importantly, there were no significant differences in the body weights of the mice between the two groups, as demonstrated in Fig. 6D. In addition, no observable toxicities were noted in the experimental animals.

At Day-4 and Day-6, 12 h following IMT1/vehicle administration, one xenograft from each group was isolated, resulting in a total of four xenografts for analysis. A portion of these xenograft tissues underwent homogenization, and subsequent analyses were performed on genes and proteins. The results revealed that IMT1 treatment did not significantly alter the expression of *POLRMT* mRNA (Fig. 6E) and protein (Fig. 6F) in pOS-1 xenograft tissues. However, there was a significant reduction in the expression of POLRMT-dependent genes, including *NDUFB8*, *UQCRC2*, and *COXI* (Fig. 6G). Further assessments indicated impaired mitochondrial functions and the presence of oxidative stress in IMT1-treated pOS-1 xenograft tissues, evidenced by increased TBAR activity (Fig. 6H), a reduced GSH/GSSG ratio (Fig. 6I), and lower ATP contents (Fig. 6J).

IMT1 administration also resulted in the inhibition of Akt-mTOR cascade activation and reduced Akt-S6K phosphorylation in pOS-1 xenograft tissues (Fig. 6K, L). Furthermore, significant activation of apoptosis was detected in IMT1-treated pOS-1 xenograft tissues. Caspase-3 activity (Fig. 6M) and Caspase-3/PARP-1 cleavage (Fig. 6N) were significantly increased following IMT1 administration in the xenograft tissues. The induction of apoptosis by IMT1 in vivo was further confirmed by an increase in TUNEL-positive nuclei in pOS-1 xenograft slides (Fig. 6O). Thus, the in vivo findings align with those observed in vitro, indicating that IMT1 administration inhibited POLRMT activity, disrupted mitochondrial functions, inhibited Akt-mTOR activation, and triggered apoptosis in pOS-1 xenografts.

## Discussion

Mitochondrial hyperfunction can significantly contribute to the growth and progression of OS and is often characterized by increased oxidative phosphorylation (OXPHOS) and energy production [11–16]. This metabolic shift not only provides cancer cells with a rapid source of ATP but also promotes the redirection of metabolic intermediates into biosynthetic pathways essential for cell proliferation [11–16]. Moreover, the metabolic intermediates generated in mitochondria can fuel the biosynthesis of macromolecules required for tumor growth [11–16]. Thus, mitochondrial hyperfunction plays a pivotal role in sustaining the rapid growth and aggressiveness of OS, making it an attractive target for potential therapeutic interventions aimed at disrupting this metabolic phenotype and inhibiting tumor progression [11–16].

**Fig. 7:**
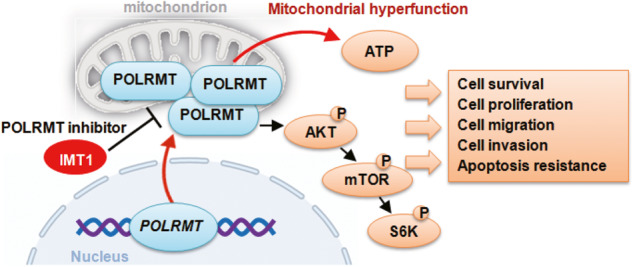
The proposed signaling carton of the present study. IMT1 suppresses POLRMT activity, impairs mitochondrial function, represses Akt-mTOR activation, and induces apoptosis, ultimately leading to the inhibition of osteosarcoma cell growth in vitro and in vivo.

The present study underscores the robust growth-inhibitory effects by IMT1 through its targeted inhibition of POLRMT. IMT1 demonstrated a profound inhibitory impact on various aspects of cellular functions, including cell survival, proliferation, cell cycle progression and migration, across both primary and immortalized OS cells. The POLRMT inhibitor-induced apoptosis in OS cells without concomitant cytotoxicity towards human osteoblasts or osteoblastic cells. IMT1 disrupted mitochondrial functions within OS cells, resulting in mitochondrial depolarization, oxidative stress, lipid peroxidation, and ATP depletion. Whereas ATP and NAC mitigated IMT1-induced cytotoxicity in OS cells. POLRMT shRNA closely recapitulated the actions of IMT1, manifesting potent anti-OS cell activity. IMT1’s efficacy was however nullified in OS cells where POLRMT had been silenced. Importantly, oral administration of IMT1 robustly suppressed the growth of OS xenografts in nude mice. In addition, IMT1 disrupted POLRMT activity, impaired mitochondrial functions, attenuated Akt-mTOR activation, and induced apoptosis within xenograft tissues (Fig. 7).

Akt-mTOR hyper-activation is frequently observed in OS cells, promoting cell survival, proliferation, motility, and resistance to apoptosis [10, 42–44, 48, 49]. This aberrant overactivation could result from genetic mutations, such as *PTEN* loss or amplification/sustained activation of growth factor receptors (i.e. EGFR), and is associated with a poor prognosis for OS patients [10, 42–44, 48, 49]. Consequently, targeting the Akt-mTOR pathway has emerged as a potential therapeutic strategy against this challenging cancer, with ongoing research focused on developing inhibitors/antibodies and combination therapies to improve treatment outcomes for OS [10, 42–44, 48, 49].

Mitochondrial hyperfunction can foster Akt-mTOR activation in cancer cells. Dysfunctional mitochondria can generate intermediates like citrate and succinate, which can activate Akt-mTOR directly or indirectly through feedback mechanisms, amplifying the signaling cascade [50–52]. The mitochondrial protein aarF domain-containing kinase 2 (ADCK2) plays a crucial role in fatty acid metabolism and coenzyme Q biosynthesis [53]. As documented by Zhang et al., the use of ADCK2 shRNA or CRISPR/Cas9-mediated knockout effectively suppressed Akt-mTOR activation in NSCLC cells [45]. Conversely, the overexpression of ADCK2 was found to enhance mitochondrial functions and activate the Akt-mTOR cascade [45]. Similarly, Han et al., demonstrated that genetic depletion of TIMM13 (translocase of inner mitochondrial membrane 13) led to mitochondrial dysfunction and the inhibition of Akt-mTOR activation in OS cells [46]. In another study by Xia et al., it was revealed that the overexpression of the mitochondrial protein YME1 Like 1 (YME1L) promoted Akt-mTOR cascade activation in NSCLC cells, while the silencing or knockout of YME1L resulted in a reduction in signaling [47].

One important finding of the present study is that IMT1 exerted a notable suppression on the activation of the Akt-mTOR cascade in OS cells. Both IMT1 treatment and POLRMT shRNA resulted in a substantial reduction in Akt1-S6K-S6 phosphorylation levels in primary OS cells. Conversely, an enhancement in phosphorylation was observed upon POLRMT overexpression. Notably, the reinstatement of Akt-mTOR activation through the introduction of caAkt1 mitigated the cytotoxic effects induced by IMT1 in OS cells. This Akt-mTOR inactivation was further corroborated in OS xenograft tissues following treatment with IMT1. Therefore, inhibition of the Akt-mTOR cascade emerges as a pivotal mechanism underlying the inhibitory effects induced by IMT1 on OS cells (Fig. 7).

### Reporting summary

Further information on research design is available in the Nature Research Reporting Summary linked to this article.

### Supplementary information

Figure S1

Reporting Summary

Author Contribution FORM

## Data Availability

All data are available upon request.
