# Supplementary figures and images for: Targeting POLRMT by a first-in-class inhibitor IMT1 inhibits osteosarcoma cell growth in vitro and in vivo
Source: Cell Death Dis. 2024 Jan 16;15(1):57. doi: 10.1038/s41419-024-06444-9 (PMC10791695; doi:10.1038/s41419-024-06444-9)

**Figure S1.**

**Figure 4.**

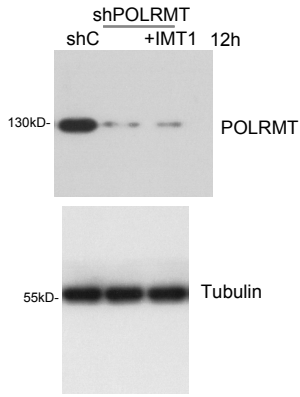

### Figure 5.

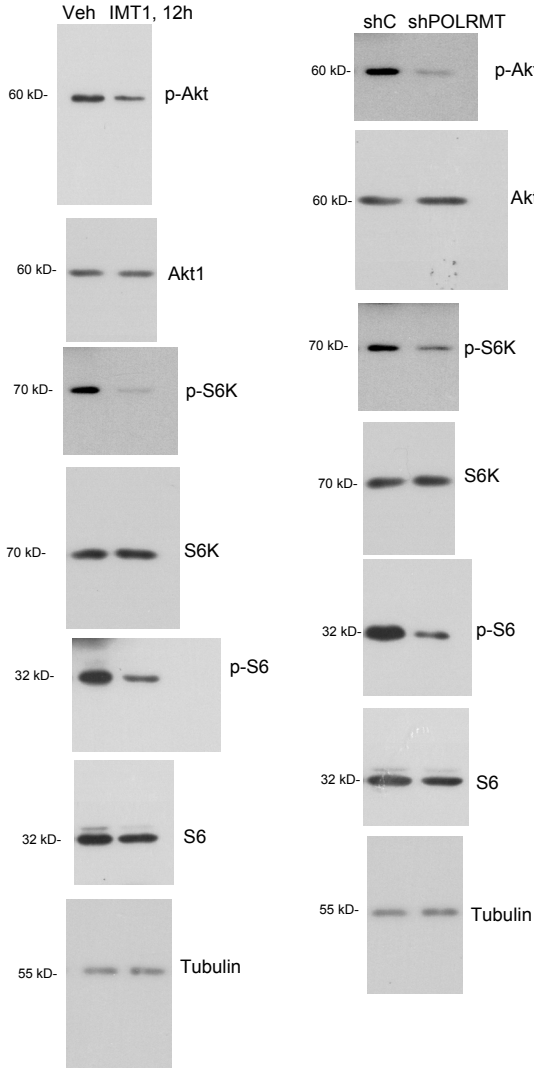

**Figure 6.**

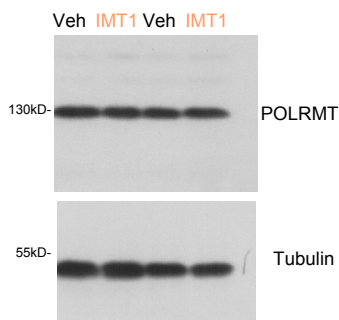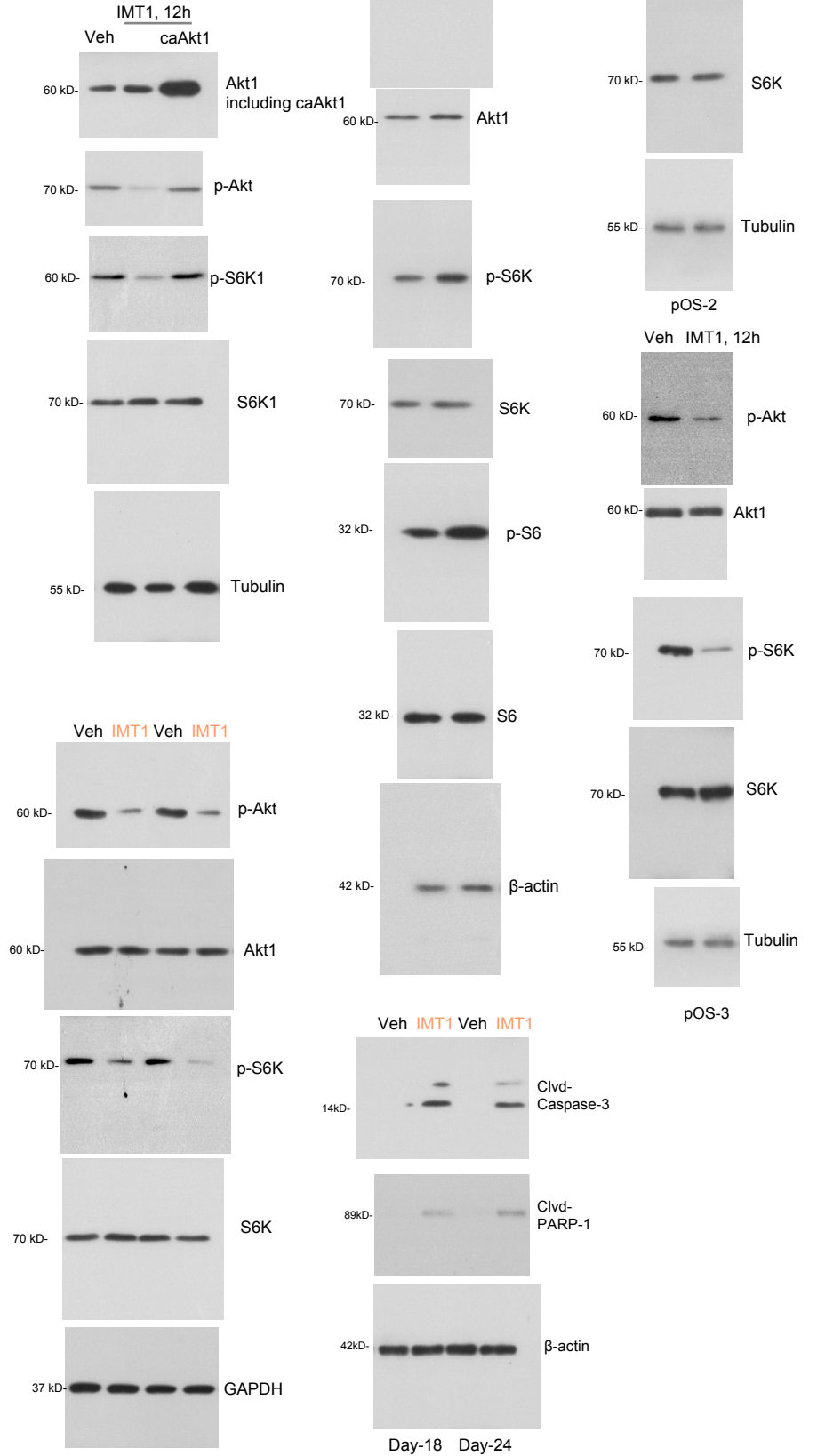

**Figure S1. The uncropped blotting images of the present study.**

Supplement: Supplementary file 1 — Figure S1 [file 41419_2024_6444_MOESM1_ESM.pdf]
