# Supplementary material for: Targeting POLRMT by a first-in-class inhibitor IMT1 inhibits osteosarcoma cell growth in vitro and in vivo
Source: Cell Death Dis. 2024 Jan 16;15(1):57. doi: 10.1038/s41419-024-06444-9 (PMC10791695; doi:10.1038/s41419-024-06444-9)
Supplement: Supplementary file 3 — Author Contribution FORM [file 41419_2024_6444_MOESM3_ESM.pdf]

DECLARATION OF CONTRIBUTIONS TO ARTICLE

ADMC

Manuscript Number: CDDIS-23-3781R

Journal Name: *Cell Death & Disease* (the 'Journal')

Proposed Title of the Contribution:

Targeting POLRMT by a first-in-class inhibitor IMT1 inhibits osteosarcoma cell growth in vitro and in vivo (the 'Contribution)

Author(s):

Yang Kong, Xiangrong Li, Huanle Zhang, Bin Fu, Hua-Ye Jian, Hui-Lin Yang and Jin Dai (the 'Authors')

For all *CDDis* articles, each person named as an author in the published version must be able to show he or she has contributed substantially to the article.

Authorship credit should be based on 1) substantial contributions to conception and design, acquisition of data, or analysis and interpretation of data; 2) drafting the article or revising it critically for important intellectual content; and 3) final approval of the version to be published. Authors should meet conditions 1, 2 and 3.

Any person who cannot be shown to have made a substantial contribution to the article cannot be listed as an author in the final version. The name of any person who is deemed to have made a minor contribution can, however, appear in the Acknowledgments section of the article.

Please complete the table below to indicate the contributions of all named authors to the manuscript.

Author Full Name:	Specification of Contribution to the Manuscript:
Yang Kong	Functional assays in osteosarcoma (OS) cells and normal obsteoblastic cells/osteoblasts, establishing OS xenografts, testing signalings and cell death/apoptosis, measuring mitochondrial functions, ROS, ATP, drafting the article and revising it, with final approval of the version submitted to the journal.
Xiangrong Li	Functional assays in osteosarcoma (OS) cells and normal obsteoblastic cells/osteoblasts, establishing OS xenografts, testing signalings and cell death/apoptosis, measuring mitochondrial functions, ROS, ATP, drafting the article and revising it, with final approval of the version submitted to the journal.
Huanle Zhang	Functional assays in osteosarcoma (OS) cells and normal obsteoblastic cells/osteoblasts, establishing OS xenografts, testing signalings and cell death/apoptosis, measuring mitochondrial functions, ROS, ATP, drafting the article and revising it, with final approval of the version submitted to the journal
Bin Fu	Functional assays in osteosarcoma (OS) cells and normal obsteoblastic cells/osteoblasts, establishing OS xenografts, testing signalings and cell death/apoptosis, measuring mitochondrial functions, ROS, drafting the article and revising it, with final approval of the version submitted to the journa.l
Hua-Ye Jian	Functional assays in osteosarcoma (OS) cells and normal obsteoblastic cells/osteoblasts, establishing OS xenografts, testing signalings and cell death/apoptosis, measuring mitochondrial functions, ROS, drafting the article and revising it, with final approval of the version submitted to the journa.l
Hui-Lin Yang	Study conception and design, and data analysis, Figure organization, involved in drafting the article and revising it critically for important intellectual content, and with final approval of the version submitted to the journal.
Jin Dai	Study conception and design, and data analysis, Figure organization, involved in drafting the article and revising it critically for important intellectual content, and with final approval of the version submitted to the journal.

Please complete the table below to indicate the contributions of all named authors to the figures.

Figure 1:

Yang Kong, Xiangrong Li, Huanle Zhang, Bin Fu, Hua-Ye Jian, Hui-Lin Yang and Jin Dai

Figure 2:

Yang Kong, Xiangrong Li, Huanle Zhang, Bin Fu, Hua-Ye Jian, Hui-Lin Yang and Jin Dai

Figure 3:

Yang Kong, Xiangrong Li, Huanle Zhang, Bin Fu, Hua-Ye Jian, Hui-Lin Yang and Jin Dai

Figure 4:

Yang Kong, Xiangrong Li, Huanle Zhang, Bin Fu, Hua-Ye Jian, Hui-Lin Yang and Jin Dai

Figure 5:

Yang Kong, Xiangrong Li, Huanle Zhang, Bin Fu, Hua-Ye Jian, Hui-Lin Yang and Jin Dai

Figure 6:

Yang Kong, Xiangrong Li, Huanle Zhang, Bin Fu, Hua-Ye Jian, Hui-Lin Yang and Jin Dai

Figure 7

Yang Kong, Xiangrong Li, Huanle Zhang, Bin Fu, Hua-Ye Jian, Hui-Lin Yang and Jin Dai

Signed for and on behalf of the Author(s):	Print Name:	Date:
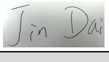	Jin Dai	12-11-2023
